# Perspectives of Japanese patients on psoriatic disease burden: Results from “Psoriasis and Beyond,” the Global Psoriatic Disease Survey

**DOI:** 10.1111/1346-8138.17424

**Published:** 2024-09-10

**Authors:** Masanori Okuse, Masayuki Soekawa, Asako Itakura, Taisuke Kawamura, Sicily Mburu, Susan Frade, Yukari Okubo

**Affiliations:** ^1^ Japan Psoriasis Association Tokyo Japan; ^2^ Novartis Pharma K.K Tokyo Japan; ^3^ IFPA Stockholm Sweden; ^4^ Novartis Pharma AG Basel Switzerland; ^5^ Department of Dermatology Tokyo Medical University Tokyo Japan

**Keywords:** awareness, comorbidities, psoriatic disease, quality of life, treatment satisfaction

## Abstract

Psoriatic disease (PsD) is a chronic disease affecting skin (psoriasis) and joints (psoriatic arthritis, PsA) that has a significant impact on patients' quality of life (QOL). We report findings from the Japanese subgroup of patients included in Psoriasis and Beyond: The Global Psoriatic Disease Survey, a cross‐sectional, quantitative online survey of patients with self‐reported, healthcare professional (HCP)‐diagnosed, moderate‐to‐severe plaque psoriasis, with or without PsA. Eligible patients who were recruited online completed a 25‐min internet‐based survey in Japanese. We assessed patients' understanding of the systemic nature of PsD, disease burden, perception towards their HCPs, treatment expectations, and satisfaction with care. Of the 148 patients, 74% were females. In total, 65% of patients were aware of the systemic nature of their disease. A minority of patients (27%) were aware that PsA was related to their psoriasis, and 30% and 42% of patients were unaware of any manifestations and comorbidities, respectively, related to PsD. Overall, 21% of patients reported that their disease has a “very large” to “extremely large” impact on their QOL (assessed by Dermatology Life Quality Index score), while the majority (61%) reported a “small” effect or “no effect” at all on QOL. Patients experienced stigma and discrimination and had a negative impact on relationships due to PsD. More patients with psoriasis and concomitant PsA (66%) were satisfied with their current treatment than those with psoriasis alone (46%). Overall, 41% of patients were not involved in deciding their treatment goals. These results suggest that Japanese patients may not be fully aware of the systemic nature of PsD, its manifestations and comorbidities. While these patients were somewhat satisfied with their current treatment, they were only occasionally consulted in deciding treatment goals. Policy measures are required to address the stigma and discrimination experienced by patients. Increased patient participation in their care supports shared decision‐making and enhanced treatment outcomes.

## 1 INTRODUCTION

Psoriasis is a chronic, systemic immune‐mediated inflammatory skin disease characterized by erythematous scaly patches or plaques of the skin with extracutaneous areas being involved, such as the nails, scalp, palms, soles, and joints.^1^ Psoriasis is associated with various systemic comorbidities such as rheumatological, cardiovascular, and psychiatric complications, which have a negative impact on the patient's quality of life (QOL).^1^, ^2^ Psoriatic arthritis (PsA) is one of the most frequent comorbidities, with 30% of the patients with psoriasis estimated to develop concomitant PsA.^1^, ^3^

Psoriatic disease (PsD), the term used to describe the various manifestations of psoriasis, PsA, and the associated comorbidities, has a substantial impact on the QOL of patients.^4^, ^5^, ^6^ In Asian countries, including Japan, patients may experience higher levels of social stigma and discrimination due to cultural and socioeconomic reasons, and therefore the impact of psoriasis on QOL may be even greater compared to non‐Asian countries.^7^

Furthermore, treatment guidelines recognize that psoriasis is a multisystem inflammatory disease and therefore emphasize a holistic approach to disease management.^8^ However, despite advances in the management of psoriasis, treatment gaps remain, which include access to biologics, access to specialists, and underestimation of severity leading to undertreatment of the disease.^9^, ^10^ Guidelines for the management of psoriasis highlight the importance of patient education and recommend that patients be directly involved in their care via shared decision‐making, which is important to facilitate comprehensive care and improve QOL.^8^

Psoriasis and Beyond: The Global Psoriatic Disease Survey was a joint research initiative between IFPA and Novartis, in collaboration with 16 patient advocacy groups, a Steering Committee comprising patient advocates, dermatology experts and rheumatology experts.^11^ The survey aimed to assess patients' understanding of the systemic nature of PsD, the related manifestations and comorbidities, and the impact of PsD on QOL.

Here, we report the findings from the Psoriasis and Beyond survey based on the perspectives of Japanese patients with psoriasis with or without PsA, and their understanding of the systemic nature of PsD and its humanistic and physical burden.

## 2 METHODS

### 2.1 Study design

The design and methodology of the Psoriasis and Beyond survey were reported in detail previously.^11^ Briefly, this was a cross‐sectional, quantitative online survey conducted in patients with plaque psoriasis with or without PsA. The survey was conducted in 20 countries across Europe, Australia, Asia Pacific, and the Americas.

Patients were recruited via online panels by the Institut de Publique Sondage d'Opinion Secteur (Ipsos SA) and the Japan Psoriasis Association (JPA), organized and coordinated by IFPA and Novartis. Following the assessment of participant's eligibility via a 5‐min online screener, a 25‐min internet‐based survey was conducted without any follow‐up. The online questionnaire was developed by Ipsos SA together with Novartis, IFPA, and the Steering Committee (Supporting Information Appendix S1). The questionnaire consisted of a combination of validated tools such as the Dermatology Life Quality Index (DLQI),^12^ the Patient Activation Measure® (PAM‐13®),^13^ the Psoriasis Epidemiology Screening Tool (PEST),^14^ and Work Productivity and Activity Impairment (WPAI).^15^ The survey was conducted in Japanese. Validated Japanese language translations of these clinical instruments were used for the questionnaire.

In this report, we present the perspectives of patients from Japan included in the global survey. Data from Japan were collected from March 30, 2021 to May 04, 2021.

### 2.2 Participants

Patients aged ≥18 years, with self‐reported, healthcare professional (HCP)‐diagnosed plaque psoriasis, with or without PsA, were included. Furthermore, patients had to have moderate‐to‐severe psoriasis (body surface area [BSA] of >5 to <10), affecting sensitive and/or prominent body parts such as face, palms, hands, fingers, genitals, soles of feet, or nails, or a BSA of ≥10 (severe) when their psoriasis was at its worst. The detailed inclusion and exclusion criteria have been previously published.^11^

### 2.3 Objectives

The primary objective was to assess patients' understanding of PsD as part of a systemic disease and the humanistic and physical burden of living with the condition. The secondary objectives were to assess patients' perceptions and attitudes related to the relationship with their HCPs, to understand the patient journey through the healthcare system, and to assess barriers to self‐management and diagnosis, patient perceptions on biologics, treatment expectations, and satisfaction with care. The detailed primary and secondary endpoints are given in Supporting Information Appendix S1.

### 2.4 Statistical analysis

All analyses were performed by Ipsos SA, and no predefined hypotheses were tested. Results were analyzed using IBM SPSS Statistic 24.0 software and reported descriptively; continuous variables were reported as mean, standard deviation, median, minimum, and maximum, while categorical variables were described in terms of frequency and percentage. Data were checked, validated, and tabulated using IBM SPSS Dimensions 2.2, a computer language designed for research data analysis. Any patients with missing values were removed from all relevant assessments (there was no imputation of missing data) but remained eligible for inclusion in other assessments.

### 2.5 Ethics

Informed consent was obtained through an electronic informed consent form from each patient after online screening and prior to participation. This study was reviewed and approved by the Institutional Ethics Committee of the Graduate School of Medicine and Faculty of Medicine, University of Tokyo. The study was conducted in accordance with legal and regulatory requirements and was consistent with the principles laid out in the Declaration of Helsinki.

## 3 RESULTS

### 3.1 Demographics

In total, 148 patients (recruited by Ipsos SA, *n* = 104; JPA, *n* = 44) from Japan were included in the Psoriasis and Beyond survey. Most of the respondents (80%) were in the age group of 18–65 years. The mean age of patients was 55.0 years for women and 56.3 years for men. Almost three‐quarters (74%) of the respondents were females. The majority of the respondents (61%) were married or in a domestic partnership and 44% had received university education.

### 3.2 Patients' understanding of psoriasis and PsA as part of a systemic disease

**FIGURE 1:**
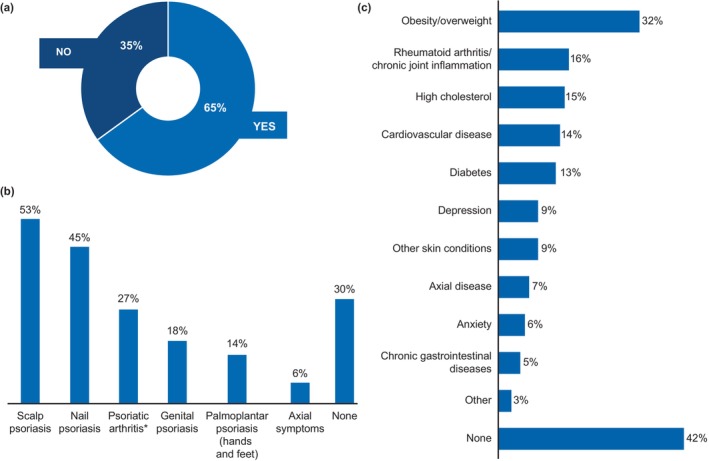
Patients' understanding of psoriasis and PsA as part of a systemic disease (*n* = 148). (a) Patients who had heard that their disease is part of a systemic disease. (b) Patients' awareness that manifestation may be related to their disease. (c) Patients' awareness that comorbidities may be related to their disease. PsA, psoriatic arthritis. *Only patients with psoriasis were questioned (*n* = 121). Data are represented as proportion of patients (%).

In total (*n* = 148), 65% of all patients were aware of the systemic nature of their disease (Figure 1a). Male respondents (74%) had a slightly higher awareness of the systemic nature of their disease compared to female respondents (62%). Patients treated with biologics (86%), other systemic medications (71%), and phototherapy (69%) had comparatively higher awareness of the systemic nature of their disease than those treated with topicals (60%). The awareness of the systemic nature of PsD was relatively consistent across different age groups, education levels, and disease severity. Awareness was higher in patients recruited via JPA (82%) than those recruited by Ipsos (58%).

### 3.3 Patients' awareness on related manifestations and comorbidities

Among all the manifestations associated with psoriasis that were listed in the survey, patients were most aware of scalp psoriasis (53%), followed by nail psoriasis (45%). Only 27% of patients with psoriasis (*n* = 121) were aware that PsA was related to their disease (Figure 1b). Furthermore, less than one‐third of the patients were aware of the relationship between their psoriasis/PsA and comorbidities; however, patients had comparatively higher awareness of the relationship between obesity (32%) and psoriasis/PsA (Figure 1c).

On average, all patients were aware of 2.3 manifestations and 2.2 comorbidities related to psoriasis or PsA. Compared to patients recruited by Ipsos SA, those recruited by the JPA had higher awareness of the disease manifestations (average, 2.2 vs. 2.6) and comorbidities (average, 1.7 vs. 3.0). The main source of awareness about manifestations and comorbidities was HCPs (53%), followed by online platforms (27%).

### 3.4 Physical impact of the disease

**FIGURE 2:**
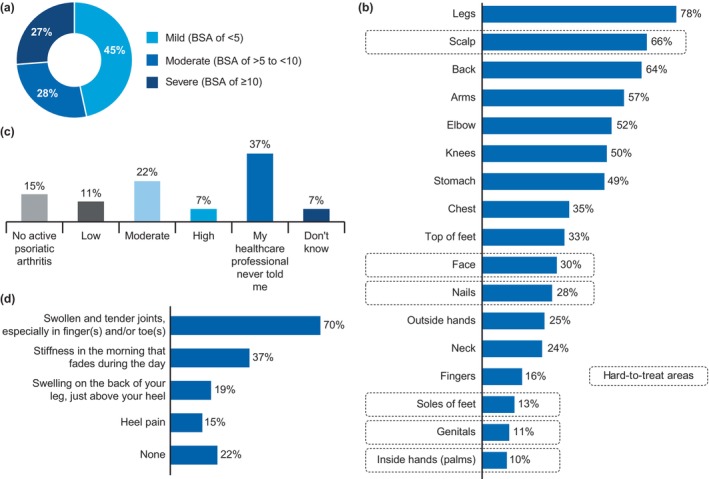
Current physical burden of psoriasis (*n* = 148) and PsA (*n* = 27). (a) Current psoriasis severity (% mild, moderate, severe). (b) Proportion of patients currently affected at hard‐to‐treat areas by current disease severity. (c) Current severity of disease in those patients with a diagnosis of PsA. (d) Current PsA symptoms. BSA, body surface area; PsA, psoriatic arthritis. Data are represented as proportion of patients (%).

Overall, 35% of patients were aware of their BSA scores, 9% did not remember, and 57% reported that their HCPs never mentioned BSA. Among patients who knew their BSA scores, about half (49%) had moderate disease severity (BSA >5 or <10) and the remaining half (51%) had severe psoriasis (BSA ≥10) when their psoriasis was at its worst. At the time of survey, 55% of patients with plaque psoriasis were suffering from moderate to severe psoriasis (Figure 2a).

On average, patients had 6.4 body parts affected by psoriasis; the most affected were legs (78%), scalp (66%), back (64%), arms (57%), and elbows (52%). Psoriasis symptoms in the average number of body parts were common in patients with even mild disease. On average, patients had 4.6, 7.0, and 8.7 body parts affected by their psoriasis in those with mild, moderate, and severe psoriasis, respectively. The most affected hard‐to‐treat areas were the scalp (66%), face (30%), and nails (28%). Other hard‐to‐treat affected areas included the soles of the feet (13%), genitals (11%), and palms (10%) (Figure 2b). It is noteworthy that 80% of patients who were on biologics (*n* = 44) had mild disease severity, whereas 20% of patients still had moderate to severe disease despite the use of biologics. Among those treated with biologics, most patients had hard‐to‐treat areas affected by psoriasis, such as the scalp (57%), nails (36%), and face (32%).

Obesity and cardiovascular disease were the most common comorbidities, diagnosed in 28% and 22% of patients, respectively. Overall, 18% (27 of 148) of all patients reported having concomitant PsA (henceforth referred to as psoriasis‐PsA). Of these, 11%, 22%, and 7% reported low, moderate, and highly active PsA, respectively, while over one‐third of patients reported that their HCPs never informed them of their PsA severity (Figure 2c). At the time of the survey, the majority of patients with PsA reported experiencing “swollen and tender joints, especially in the finger(s) and/or toe(s)” (70%), and more than one‐third of the patients reported “stiffness in the morning that fades during the day” (Figure 2d).

Using PEST (a validated 5‐item questionnaire to help identify PsA at an early stage), among patients (*n* = 121) who had not previously been diagnosed with PsA, 7% screened positive for PsA (6% of patients with mild psoriasis and 8% of patients with moderate or severe psoriasis). Out of all psoriasis‐only patients who experienced any joint symptoms in the previous year, 41% of patients reported to have asked their HCPs about their joint symptoms.

### 3.5 Impact of the disease on life

**FIGURE 3:**
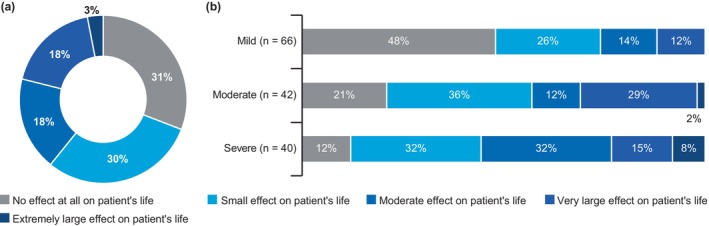
Effect of psoriasis on patients' QOL (*n* = 148). (a) Effect on patients' QOL. (b) Impact on QOL based on current disease severity. QOL, quality of life. Data are represented as proportion of patients (%).

On DLQI assessment, 21% of patients (*n* = 148) reported that their skin problems had a “very large” to “extremely large” impact on their QOL, whereas the majority of the patients (61%) reported either a small effect or “no effect at all” on their QOL (Figure 3a).

The impact of skin problems on QOL was greater in patients with moderate and severe psoriasis than in patients with mild psoriasis (Figure 3b). The “very large” to “extremely large” impact of disease on QOL was higher in patients with at least one hard‐to‐treat area affected by psoriasis (22%) than in patients with no hard‐to‐treat area involvement (16%). Half the patients (50%) using biologics (*n* = 44) reported that skin problems had no impact on their QOL over the previous week. Furthermore, 43% of patients with depression and anxiety reported a very large to extremely large effect of their psoriasis symptoms on their QOL.

Using WPAI, among those who were able to attend work (*n* = 115) despite having skin problems, 26% expressed that their skin problems impacted their work “a little” and 5% reported that skin problems impacted their work “a lot.” Furthermore, of all patients (*n* = 148), 18% reported that their career or choice of work was influenced by their disease, 11% experienced discrimination at work, and 10% faced difficulty in finding a job due to their psoriasis or psoriasis‐PsA. In addition, 9% of patients reported that their skin problems prevented them from working or studying over the previous week.

On average, patients reported that their disease had an impact of 3.7 (on a scale of 0–10) on their ability to perform daily activities. Overall, 20% of patients (*n* = 148) reported that their health problems had no impact, whereas 5% of patients reported that their health problems completely prevented them from performing their daily activities. Among patients treated with biologics (*n* = 44), 50% reported that their health problems had little to no effect on their ability to perform daily activities during the past week.

**FIGURE 4:**
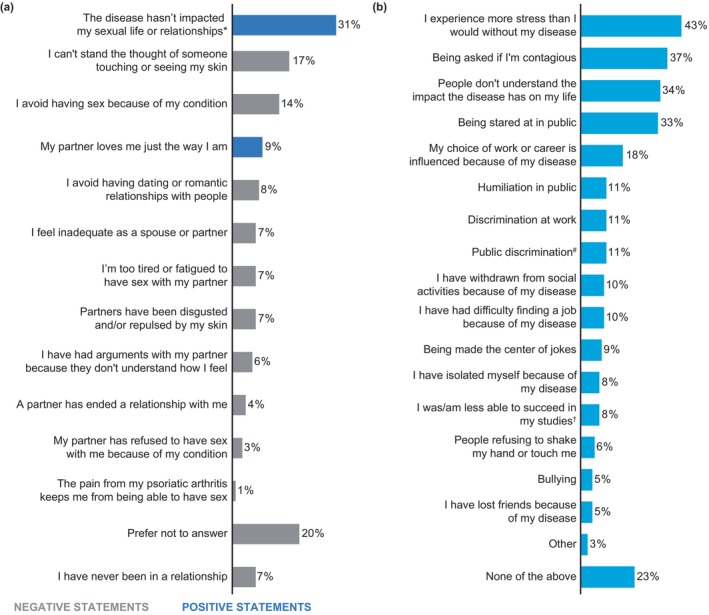
Effect of psoriatic disease on personal relationships and experiences of discrimination (*n* = 148). (a) Impact on sexual life and relationships. (b) Experiences of discrimination and stigma. *With my current/previous spouse or partner. ^#^Refusal to give me a treatment at beauty clinic/cosmetic studio, people refusing to serve me in shops, being asked to leave a form of public transport. ^†^Than I would be without my disease. Data are represented as proportion of patients (%).

On average, patients rated their emotional well‐being as 6.5 on a scale of 1 to 10 (1 = no impact at all on overall emotional well‐being; 10 = impacts overall emotional well‐being very much). Psoriasis/psoriasis‐PsA impacted the overall emotional well‐being very much in 39% of patients. Living with psoriasis or psoriasis‐PsA made patients feel ashamed of their skin (53%), ashamed of their body (40%), and depressed and helpless (36%). The impact of psoriatic disease on sexual life and relationships and their experience of stigma and discrimination is shown in Figure 4a,b. Overall, 31% reported no impact on their sexual life and relationship due to their condition, whereas 17% said they could not stand the thought of someone touching or seeing their skin, and 14% avoided having sex because of their condition. Furthermore, 43% of patients with psoriasis or psoriasis‐PsA reported that they experienced more stress than they would without the disease, 37% had been asked if they were contagious, 34% felt that people do not understand the impact of disease on patient's life, and 33% reported that they were stared at in public.

**FIGURE 5:**
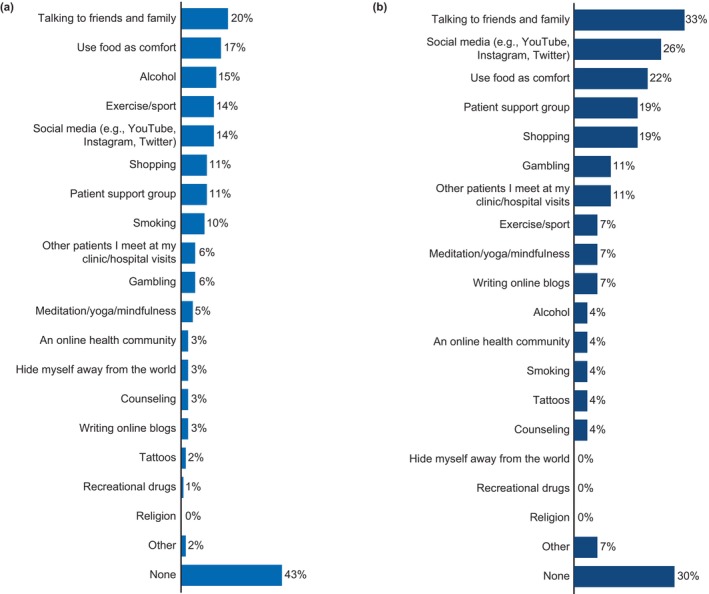
Coping mechanisms used by psoriasis (*n* = 148) and psoriasis‐PsA patients (*n* = 27). (a) Proportion of patients with psoriasis (%) using specified coping mechanisms to deal with their disease. (b) Proportion of patients with psoriasis‐PsA (%) using specified coping mechanisms to deal with their disease. PsA, psoriatic arthritis. Data are represented as proportion of patients (%).

Coping mechanisms used by all patients are shown in Figure 5a,b. On average, patients with psoriasis used 2.6 coping strategies and those with psoriasis‐PsA used 2.7 coping strategies to deal with their condition. Talking to friends and family was the most common coping mechanism used by patients with psoriasis (20%) and psoriasis‐PsA (33%).

Patients' knowledge, skills, and confidence to manage their own health were assessed using PAM®, a 13‐item survey that characterizes patients into one of four activation levels. Of all patients (*n* = 148), 57% said they were disengaged and overwhelmed (level 1), 20% were becoming aware but still struggling (level 2), 16% were taking action (level 3), and only 1% were maintaining behaviors and pushing further (level 4).

### 3.6 Patient life experience through the healthcare system

Patients first experienced symptoms of plaque psoriasis at the mean age of 33.6 years and were diagnosed with the condition at the mean age of 36.2 years (diagnostic delay of 2.6 years). In the case of psoriasis‐PsA, the mean ages of first symptom onset and diagnosis of PsA were 41.2 and 44.4 years, respectively. This demonstrates a delay in PsA diagnosis of 3.2 years. The diagnostic delay was longer in male patients with both psoriasis and psoriasis‐PsA (3.7 and 5.4 years, respectively) compared to their female counterparts (2.2 and 2.0 years, respectively).

Dermatologists confirmed the diagnosis of psoriasis and PsA in 93% and 70% of the cases, respectively. The majority of the patients with psoriasis were primarily treated by a dermatologist (89%), followed by general physician or family practitioner (6%). Patients with psoriasis‐PsA (*n* = 27) were primarily managed by rheumatologists (30%) and general physicians (30%) followed by dermatologists (26%).

**FIGURE 6:**
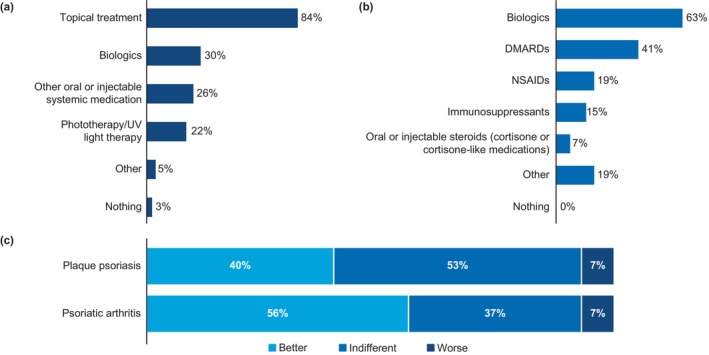
Current treatments and effect of treatments for psoriasis (*n* = 148) and PsA (*n* = 27). (a) Proportion of patients (%) receiving specified treatments to treat psoriasis. (b) Proportion of patients (%) receiving specified treatments to treat PsA. (c) Effect of the current treatments on psoriasis and PsA. DMARD, disease‐modifying antirheumatic drug; NSAID, nonsteroidal anti‐inflammatory drug; PsA, psoriatic arthritis; UV, ultraviolet. Data are represented as proportion of patients (%).

The most common treatments used by patients with psoriasis were topical treatment (84%), followed by biologics (30%) and other oral or injectable systemic medication (26%; Figure 6a). On average, patients with psoriasis received 4.3 topical treatments, 0.6 biologics, and 1.1 other oral or injectable systemic medications. By severity, 53%, 12%, and 10% of patients with mild, moderate, and severe psoriasis, respectively, received biologics. In patients with psoriasis‐PsA (*n* = 27), the most common current treatment was biologics (63%), followed by disease‐modifying antirheumatic drugs (DMARDs; 41%), nonsteroidal anti‐inflammatory drugs (NSAIDs; 19%), and immunosuppressants (15%) (Figure 6b). On average, patients with psoriasis‐PsA received 1.7 biologics, 1.0 DMARDs, and 1.5 NSAIDs.

Only 40% of patients with psoriasis and 56% of patients with psoriasis‐PsA reported an improvement in their condition with the current treatment; 53% and 37%, respectively, reported that their condition was indifferent (Figure 6c). Nearly a quarter (24%) of the patients with psoriasis had clear skin with the current treatment and 46% believed in the possibility of achieving clear or almost clear skin.

### 3.7 Patients' satisfaction with care, perception of biologics, and self‐management

**FIGURE 7:**
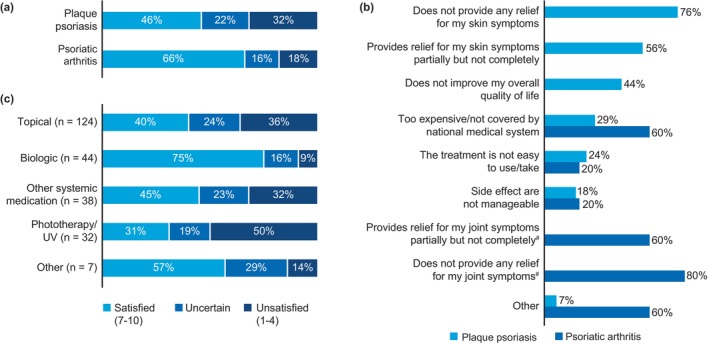
Treatment satisfaction overall and based on current treatment. (a) Proportion of patients (%) with psoriasis and psoriasis‐PsA satisfied with their current treatment. (b) Frequency (%) of key reasons cited for dissatisfaction with current treatment regimen*. (c) Patient satisfaction based on current treatments in patients with psoriasis. PsA, psoriatic arthritis; UV, ultraviolet. *Patients were allowed to select more than one reason for dissatisfaction. ^#^Questioned only to patients with psoriasis‐PsA. Data are represented as proportion of patients (%).

More patients with psoriasis‐PsA (66%) were satisfied with their current treatment than those with psoriasis (46%; Figure 7a). The main reasons for dissatisfaction in patients with psoriasis were no relief of skin symptoms at all (76%), only partial relief of skin symptoms (56%), and no improvement in QOL (44%; Figure 7b).

The level of satisfaction with current treatment varied based on the severity of psoriasis. Among patients with moderate and severe psoriasis, 46% and 53%, respectively, reported being unsatisfied, whereas only 9% of patients with mild psoriasis were unsatisfied with the current treatment. Treatment satisfaction was the highest in patients currently receiving biologics (75%; Figure 7c) and among those recruited by the JPA (73%). Overall, 13% of patients had ever refused a biologic. Among those who did so, the main reasons for refusing a biologic treatment were costs (79%), fear of possible side effects (47%), and/or frequency of treatment (32%).

Only 14% of all patients with psoriasis (*n* = 121) who did not have a PsA diagnosis recalled being advised to monitor their joints for symptoms such as joint pain, heel pain, or stiffness. Of those patients, almost one in four did not check their joints whatsoever, one in four reported that they checked their joints for symptoms at least once a month, and over one in four patients checked their joints less than once a year.

### 3.8 Patients' perceptions and attitudes related to the relationship with their HCPs

**FIGURE 8:**
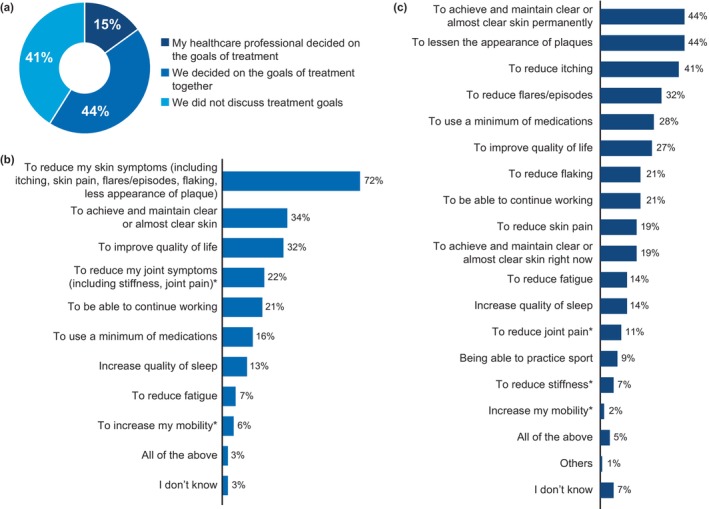
Treatment goals. (a) Proportion of all patients, patients with psoriasis only, and patients with psoriasis‐PsA involved in deciding treatment goals along with their HCP. (b) Treatment goals aligned with HCPs. (c) Personal treatment goals described by patients. *Only patients with psoriasis‐PsA were questioned. HCP, healthcare professional; PsA, psoriatic arthritis. Data are represented as proportion of patients (%).

Most patients were confident in the abilities of their HCPs to treat their joints (81%) and skin (57%), 68% always followed the advice of their HCPs, and 66% felt their HCPs were clear with the information about treating their conditions. Furthermore, around two‐thirds of the patients (63%) felt listened to by their HCPs and less than half (49%) felt their HCPs fully understood the impact of their conditions on their everyday lives. Only 46% felt that they could get in touch with their HCPs when in need. Nonetheless, only 44% discussed their treatment goals with their HCPs, whereas 15% of the patients reported that their HCPs decided on the treatment goals and 41% of patients reported that they never discussed treatment goals with their HCPs (Figure 8a). The main treatment goal for HCPs as well as patients was to reduce the skin symptoms and to achieve and maintain clear or almost clear skin. Treatment goals aligned with HCPs for patients with psoriasis and psoriasis‐PsA are shown in Figure 8b. In addition, patients' personal treatment goals are shown in Figure 8c.

Only 31% of all patients (*n* = 148) reported that their HCP actively explained the association between plaque psoriasis and PsA, whereas 39% of patients reported that their HCP never mentioned this link. Only 29% of all patients with psoriasis‐only (*n* = 121) stated that their HCPs had asked them if they ever experienced PsA symptoms such as swollen and tender joints, especially in the finger(s) and/or toe(s).

## 4 DISCUSSION

This report is based on perspectives from Japanese participants in the global Psoriasis and Beyond survey, which evaluated patients' understanding of the systemic nature of their PsD and the humanistic and physical burden of living with this condition.^11^ The results from the survey highlight that a fair proportion of patients lack awareness of the systemic nature of PsD (35%) and its associated manifestations (30%) and comorbidities (42%). The findings also demonstrate a substantial impact of PsD on the humanistic and physical burden of living with this condition.

This survey had a higher percentage of female participants, which contrasts with the prevalence of psoriasis among female patients in Japan.^16^ The survey found that in Japan, most patients were aware of one or more manifestations (70%) as well as comorbidities (58%) related to their disease; however, this awareness was generally lower than that in the global population.^11^ Interestingly, patients recruited by the JPA were more educated and had a higher level of awareness about their disease compared to patients recruited by Ipsos SA. In Japan, the level of awareness regarding the systemic nature of PsD (65%) was comparable to the global data (69%).^11^ These results suggest that patients need to be educated more about the systemic nature of the disease.

Overall, 18% of patients had a confirmed diagnosis of PsA, which is lower compared to the global survey (30%)^11^ and general population (up to 30%).^3^ In addition, the diagnostic delay for PsA was 3.2 years in Japan, which is greater than the average delay of 2 years in the global population.^11^ Furthermore, 7% of the patients with psoriasis only screened positive for PEST. These results highlight the need for active screening for PsA by their psoriasis‐treating HCPs.

Among patients with psoriasis, nearly one‐third (31%) had heard about the link between PsA and psoriasis from their treating HCPs. Furthermore, 27% of all respondents were aware that PsA was related to their psoriasis. This suggests that patients are not provided with sufficient information related to their condition, and therefore HCPs have an important role in educating patients about the risk of developing PsA and comorbidities, considering that most patients believed in their HCPs' abilities and followed their advice. Furthermore, there is a need to educate HCPs to actively inform their patients regarding their disease and related conditions to increase awareness. The academic societies should also focus on educating other related specialists (rheumatologists and orthopedics) along with dermatologists.

While most respondents had mild psoriasis (45%) at the time of survey, based on the standard definition of BSA <5, their description of current symptoms and body parts affected, comorbidities, and the percentage of patients on biologics suggests that a greater number of patients experienced more severe psoriasis. Similar findings were also highlighted in both the MAPP and UPLIFT surveys.^17^, ^18^ Therefore, to avoid underestimating the severity of the disease and thereby undertreating the condition, various aspects such as symptoms, body parts affected, and impact of psoriasis on QOL should be taken into consideration in addition to BSA.

This survey showed that a majority of the patients with psoriasis who experienced joint symptoms (59%) never discussed these with their HCP. However, this percentage is lower compared than the results from UPLIFT study, in which almost three‐quarter of patients never had this discussion.^19^ Furthermore, there were considerable gaps in knowledge about PsA severity, with more than one‐third of the patients being uninformed about their PsA severity and the relationship between psoriasis and PsA by their HCP. These results highlight the gap in patient‐HCP engagement, therefore increased patient‐HCP engagement may result in increased awareness among patients, leading to better outcomes and treatment satisfaction.

The impact of psoriasis on QOL was lower in Japan compared to the global population. The percentage of patients with “moderate effect” to “extremely large effect” of psoriasis on QOL was 39% in Japan compared to 69% in the global population.^11^ This contrasts with the larger proportion of patients who had moderate to severe disease in Japan compared to global population (55% vs. 40%). Although the impact of disease on QOL was lower in Japan compared to the global population,^11^ the results from this subgroup are in line with the previously reported impact of psoriasis on QOL in Japanese patients.^19^ This suggests that the impact of psoriasis on QOL was generally lower, which is reflected in the personal treatment goals of Japanese patients, in which only 27% considered QOL improvement as part of their treatment goals. However, in Japan, 43% of patients with psoriasis involvement in any of the hard‐to‐treat areas had a moderate to extremely large effect of disease on their QOL, comparable to the results from the Japanese cohort in the UPLIFT survey, in which nearly half of the patients with psoriasis involvement in at least one hard‐to‐treat area experienced at least a moderate impact on QOL.^19^ This suggests that psoriasis of hard‐to‐treat areas is extremely distressing. Furthermore, it was previously shown that psoriasis severity negatively correlated with work productivity in Japanese patients with psoriasis. Psoriatic lesions on the hands and PsA comorbidity were significantly associated with health‐related work productivity loss. Therefore, patients with severe psoriasis, especially with hand involvement or PsA, should be actively treated.^20^

In this survey, patients with psoriasis or psoriasis‐PsA experienced a substantial impact on their emotional well‐being, with 39% of patients having reported that their disease impacted emotional well‐being very much. More than one‐third (43%) of the patients with psoriasis experiencing anxiety and depression reported a very large to extremely large effect on their QOL. These findings suggest that in order to fully address the condition, clinicians need to follow a holistic approach that focuses on psychological well‐being along with the physical and social well‐being.^21^, ^22^

PAM® results show that only 1% of patients were at level 4 (highest level of activation), substantially lower compared to the global population in which one‐fifth of the patients were at level 4.^11^ Furthermore, more than half of the patients were at level 1, which is substantially higher compared to those at the same level of activation in the global population (9%).^11^ These results indicate that patients in Japan may lack awareness off the importance of their role in managing their own health. Furthermore, Japanese patients have confidence in the abilities of their HCPs and follow their directions for managing their PsD. However, enhancing self‐management skills in Japanese patients might contribute to improved clinical outcomes and QOL.

The majority of patients with psoriasis‐PsA (81%) had trust in the abilities of their HCPs to treat their joint symptoms, whereas only 57% of all patients were confident of the abilities of their treating HCPs. Despite a large number of patients expressing a high level of trust in their psoriasis‐treating HCPs, only 44% of patients were involved in goal setting with their HCPs, whereas 41% of respondents stated that they never discussed treatment goals with their HCPs. This is important because treatment goals may differ between HCPs and patients.^19^, ^23^ In Japan, the personal treatment goals of the majority of the patients are focused on achieving and maintaining clear skin, reducing physical impact of disease, and using minimum medication. Therefore, it is important for HCPs to consider individual patient's goals before initiating treatment.^23^, ^24^, ^25^

In Japan, only 24% of patients achieved clear skin with current treatment compared to 52% in the global survey.^11^ However, the percentage of patients who believed in the possibility of a clear skin was 46%, higher than those who actually achieved it. This indicates higher treatment expectations in this patient population. Whether improved patient access to biologics could minimize the gap remains to be investigated. Most patients treated with biologics experienced a better QOL in Japan and a high level of treatment satisfaction. Patients with psoriasis‐PsA (66%) were more satisfied with their current treatment than those with psoriasis only (46%). This is an interesting finding because PsA symptoms are usually harder to treat.^26^ However, this needs to be further investigated in a larger population. A potential explanation for the high satisfaction in patients with psoriasis‐PsA could be the high proportion of patients with PsA receiving biologics. Another explanation for the difference in treatment satisfaction levels between patients with psoriasis and those with psoriasis‐PsA could be due to the difficulty in eliminating skin symptoms in patients with psoriasis only as compared to slowing down the disease progression in patients with PsA, leading to higher satisfaction.

This survey, as with others, is likely to have limitations. The results were based on patients' experience of disease, which might influence the results. The survey included patient's self‐reported data and therefore recall bias may be a possibility. The patient's current disease severity and their emotional state at the time of the survey may also influence their survey responses. Moreover, patients with bothersome or uncontrolled disease may have been more likely to participate, leading to over‐representation of patients with more severe disease.

In conclusion, this analysis suggests that Japanese patients with self‐reported physician‐diagnosed psoriasis or psoriasis‐PsA are not fully aware of the systemic nature of their psoriatic disease, its manifestations, and comorbidities. HCPs and patient organizations have a crucial role in promoting awareness and education. Furthermore, patients with psoriasis should be encouraged to collaborate with patient organizations, which may contribute to increased awareness. The findings suggest that physical impact alone does not contribute to overall disease severity. Furthermore, assessment of disease severity and its impact should be based on a holistic approach, including physical impact, mental and emotional well‐being, current symptoms, comorbidities, involvement of hard‐to‐treat areas, and impact on QOL. Since previously undiagnosed patients were screened positive for PsA during the survey, HCPs treating patients with psoriasis should be encouraged to actively screen for PsA using PEST. This could lead to reduced diagnostic delay for patients with psoriasis with undiagnosed PsA in Japan. Patients who experience a substantial impact on mental and emotional well‐being could be effectively managed using a multidisciplinary approach. Furthermore, the findings from this survey show that the majority of Japanese patients with psoriasis (with or without PsA) experienced discrimination and stigmatization, which requires to be addressed through policy measures. Increased patient participation in their care facilitates shared decision‐making and could support realistic goal setting and treatment expectations, thereby resulting in improved patient outcomes.

## CONFLICT OF INTEREST STATEMENT

Masanori Okuse: Member of the Patient Engagement Advisory Think Tank organized by Novartis, patient advisor to PhRMA Japan, board member of IFPA. Masayuki Soekawa: Patient advisor to Boehringer Ingelheim. Asako Itakura: Employee of Novartis. Taisuke Kawamura: Employee of Novartis. Sicily Mburu MD, MSc Epi.: No individual conflict of interest, employee of IFPA. Dr. Mburu: Has participated in consulting on behalf of IFPA speaking, advisory boards, research and organizing conference but has not received payment for this directly. IFPA is supported by grants and funding from AbbVie, Amgen, Bristol Myers Squibb, Boehringer Ingelheim, Janssen, Leo Pharma, Eli Lilly, Novartis, Pfizer, UCB and Takeda. Susan Frade: Employee of Novartis. Yukari Okubo: Received research grants from AbbVie, Eisai, Maruho, Shiseido, Sun Pharma, and Torii Pharmaceutical; has received honoraria from AbbVie, Amgen, Boehringer Ingelheim, Bristol Myers Squibb, Eisai, Eli Lilly, Janssen Pharma, Jimro, Kyowa Kirin, Leo Pharma, Maruho, Novartis Pharma, Pfizer, Sanofi, Sun Pharma, Taiho Pharmaceutical, Tanabe Mitsubishi, Torii Pharmaceutical, and UCB; has conducted clinical trials for AbbVie, Amgen, Boehringer Ingelheim, Bristol Myers Squibb, Eli Lilly, Janssen, Leo Pharma, Maruho, Pfizer, Sun Pharma, and UCB.

## Supporting information


Appendix S1.
