# Supplementary material for: Perspectives of Japanese patients on psoriatic disease burden: Results from “Psoriasis and Beyond,” the Global Psoriatic Disease Survey
Source: J Dermatol. 2024 Sep 10;51(10):1298–309. doi: 10.1111/1346-8138.17424 (PMC11484140; doi:10.1111/1346-8138.17424)
Supplement: Supplementary file 1 — Appendix S1. [file JDE-51--s001.docx]

# **Appendix**

## Exclusion criteria

Any patient who had participated in another survey regarding psoriasis or psoriatic arthritis (PsA) within the previous 4 weeks or had worked for market research institutes/pharmaceutical companies were excluded. Further respondents who were not in agreement with the general pharmacovigilance terms and conditions were excluded.

## Questionnaire

A combination of validated tools such as the Dermatology Life Quality Index (DLQI)^1^, Patient Activation Measure^®^ 13 (PAM-13^®^),^2^ and the Work Productivity and Activity Impairment (WPAI)^3^ were included in the questionnaire. The remainder of the questionnaire was made up of questions that were previously used in the *Clear About Psoriasis* global survey^4^ to allow comparability, as well as newly defined questions (tailored to the objectives of the current survey). The Steering Committee reviewed all the questions for appropriateness that were excluded from the validated questionnaires. This survey was translated into the local language. Further, available validated translations of the validated clinical instruments were used where possible.

## Primary endpoints

The primary objective was determined based on the following sections of the questionnaire:

- Awareness of manifestations and comorbidities of psoriatic disease (PsD)
- Awareness and understanding of the term “PsD”
- Physical impact of the disease
- Impact of the disease on work experiences and everyday experiences (including social and personal life, self-esteem, and emotional well-being)

Key secondary objectives were explored through the following endpoints:

1. *To assess the patient's perceptions and attitudes related to the relationship with their physician*
   1. The patient's view on whether their healthcare professional (HCP) fully understands how much their disease and treatments are affecting their quality of life (QOL)
   2. Whether patients feel listened to by their HCP
   3. The patient’s role in deciding their treatment goals and treatment plan
   4. Whether and how the patient has been engaged by their HCP in a conversation about psoriasis as more than skin, including the manifestations and comorbidities
   5. Patients’ subjective competence and trust in their HCP
   6. Patient and HCP-perceived goals of treatment, and the associated levels of alignment/misalignment in these goals
2. *Understand the patient journey to diagnosis and through the healthcare system*
   1. Time between first symptoms and diagnosis
   2. Physician specialty currently treating disease
   3. Frequency of physician visits
   4. Number of treatments by class currently taken and total treatments taken up to date
   5. Impact of treatment on symptoms
   6. Satisfaction with current treatment
   7. Awareness of the possibility of achieving clear skin among patients with psoriasis
   8. Coping mechanisms
   9. Patient’s level of knowledge, skills, and confidence to manage their disease using
      PAM-13^®^
   10. Sources of information related to PsD
3. *Assess barriers to self-management, barriers to diagnosis, patient perceptions on biologics, treatment expectations, and satisfaction with care*
   1. Levels of patient-reported satisfaction with disease control/treatment and reasons for dissatisfaction
   2. Subjective feelings of satisfaction with care
   3. Assessment of expectations of care
   4. Reasons for refusal of a biologic treatment
   5. Engagement in self-screening for PsA among patients with psoriasis
4. *Other variables of interest*
   1. Sociodemographic characteristics (age, gender, income, place of living, occupation status, and civil status)
   2. Self-reported diagnosis on psoriasis and/or PsA
   3. Self-reported assessment of severity of the psoriasis and/or PsA
   4. Self-reported diagnosis of comorbidities
   5. Financial impact of the disease

**Supplementary References**

1. Hongbo Y, Thomas CL, Harrison MA, Salek MS, Finlay AY. Translating the science of quality of life into practice: What do dermatology life quality index scores mean? *J Invest Dermatol*. 2005;125(4):659–64.

2. Hibbard JH, Mahoney ER, Stockard J, Tusler M. Development and testing of a short form of the patient activation measure. *Health Serv Res*. 2005;40(6 Pt 1):1918–30.

3. Mease PJ, Palmer JB, Hur P, Strober BE, Lebwohl M, Karki C, et al. Utilization of the validated Psoriasis Epidemiology Screening Tool to identify signs and symptoms of psoriatic arthritis among those with psoriasis: a cross-sectional analysis from the US-based Corrona Psoriasis Registry. *J Eur Acad Dermatol Venereol*. 2019;33(5):886–92.

4. Armstrong A, Jarvis S, Boehncke WH, Rajagopalan M, Fernandez-Penas P, Romiti R, et al. Patient perceptions of clear/almost clear skin in moderate-to-severe plaque psoriasis: results of the Clear About Psoriasis worldwide survey. *J Eur Acad Dermatol Venereol*. 2018;32(12):2200–7.
